# First-Year University Students Who Self-Select into Health Studies Have More Desirable Health Measures and Behaviors at Baseline but Experience Similar Changes Compared to Non-Self-Selected Students

**DOI:** 10.3390/nu10030362

**Published:** 2018-03-16

**Authors:** Mary-Jon Ludy, Abigail P. Crum, Carmen A. Young, Amy L. Morgan, Robin M. Tucker

**Affiliations:** 1 Department of Public & Allied Health, Bowling Green State University, Health & Human Services, Bowling Green, OH 43403, USA; mludy@bgsu.edu (M.-J.L.); apcrum@bgsu.edu (A.P.C.); 2 Department of Athletics, Rutgers University, 1 Scarlet Knight Way, Piscataway, NJ 08854, USA; cyoung@scarletknights.com; 3 School of Human Movement, Sport, & Leisure Studies, Bowling Green State University, 216 Eppler South, Bowling Green, OH 43403, USA; amorgan@bgsu.edu; 4 Department of Food Science and Human Nutrition, Michigan State University, 2110 S. Anthony Hall, 474 S. Shaw Ln, East Lansing, MI 48824, USA

**Keywords:** alcohol, BMI, college health, college students, diet, health behaviors, risk assessment, self-selection, weight gain, young adults

## Abstract

Studies demonstrate that first-year university students are at high risk for weight gain. These reports typically rely on self-selected participants. The purpose of this study was to explore if students who chose to participate in a health-based research study had more desirable health measures and behaviors than students who completed health assessments as part of a first-year seminar course. Health measures included blood pressure (BP), body mass index (BMI), and percent body fat. Health behaviors included dietary patterns (Starting the Conversation questionnaire) and alcohol use (Alcohol Use Disorders Identification Test-Consumption). A total of 191 (77% female) participants completed testing in the self-selected “Health Study” group, whereas 73 of the 91 students (80%, 55% female) enrolled in the “Seminar” allowed their data to be used for research purposes. Baseline measures favored Health Study participants, including but not limited to fewer participants with undesirable BMI (≥25.0 kg/m^2^; males and females) and a smaller percentage of participants with undesirable BP (systolic ≥120 mmHg and/or diastolic ≥80 mmHg; females only). Differences in dietary behaviors at baseline were inconsistent, but Seminar students engaged in more problematic alcohol-use behaviors. While both groups experienced undesirable changes in health measures over time, the degree of change did not differ between groups. Changes in health behaviors over time typically resulted in undesirable changes in the Seminar group, but the magnitude of change over time did not differ between groups. Thus, results from first-year university students who self-select into health studies likely underestimate the seriousness of undesirable health measures and behaviors but may accurately reflect the degree of change over time.

## 1. Introduction

Numerous studies suggest that first-year university students are at high risk for weight gain (recently reviewed by [1,2]). Meta-analyses conclude that weight gain during the first year of university is approximately 1.6–1.8 kg [1,2]. These estimates agree with another large-scale study of self-reported weight gain, where female first-year students reported gaining 1.4 kg and males reported gaining 1.6 kg (*N* = 4687) [3]. Further, other studies indicate that weight gain can occur early in the first year, even during the first semester [4,5]. While gains are typically highest in the first year [6,7], this weight gain continues throughout the university experience [1,6,7,8,9], and those who enter their first year of university already overweight or obese are at higher risk for developing chronic diseases than their lean counterparts [10]. Even more concerning, it could be the case that these reports underestimate the negative health outcomes associated with the university experience due to self-selection bias.

Since participation is typically voluntary in studies measuring weight and health changes among university students, participant self-selection by healthier individuals could pose a problem [11,12,13,14]. Self-selection bias hampers the generalizability of reported findings to the larger population of interest—regardless of sample size—because the study population differs systematically from the group it is meant to represent [15,16,17]. It could be that students who choose to participate in longitudinal investigations related to weight and health changes do not represent the larger population of university students, as participants may be more knowledgeable about and engaged in positive health behaviors. If this is the case, it is likely that weight and fat gains are higher among the general population of university students given previous work that suggests individuals with higher measures of adiposity are more likely to choose not to participate in or to drop out of health studies [18,19,20].

Alternatively, there could be reasons other than an interest in personal health that motivate university students to participate in research studies on this topic. Students new to campus might be interested in participating in a research study because they can earn credit for a class [21,22], help society [23], or because of financial incentives [24,25]. In the case of financial incentives, a number of studies suggest that payment was a leading factor in participants deciding to take part in a health study (reviewed by [26]). If such factors are more salient motivators for study involvement than health-related interests, these participants might not be substantively different from their peers in terms of general health measures.

The purpose of this study was to explore whether first-year students who chose to participate in a health-based research study had more desirable health measures and behaviors than students who completed health assessments as part of a first-year seminar course at both the beginning of the academic year (August) and at the end of the first semester (December) and to assess the degree of change over time between the two groups. We hypothesized that self-selected health study participants would have more desirable health measures (blood pressure (BP), body mass index (BMI), percent body fat (%BF)), engage in fewer negative health behaviors (unhealthy diet and alcohol use patterns), and that these participants would experience undesirable changes in these parameters to a lesser degree over time than participants in a seminar course.

## 2. Materials and Methods

### 2.1. Study Design

Participants for this study were recruited from two pools. First, students who had an interest in participating in an on-going (Fall 2012–Fall 2015) study examining health and health behaviors among first-year university students (Health Study) were recruited through informational flyers, emails, and social media. Participation required contacting the researchers, making an appointment, and reporting to the laboratory at the beginning and end of the semester in which they participated in the study. Participants were compensated $5 in campus cash or a spirit item (e.g., water bottle or t-shirt) at each visit. Informed consent was obtained prior to study participation. The second pool of students completed required health assessments as part of a graded 1-credit hour seminar course designed to assist with the transition to university during their first semester (Fall 2016) and chose to share their data for research purposes (Seminar). Sharing of the data was voluntary, and participation had no effect on the course grade; researchers did not know who consented until after grades were submitted. Seminar students were enrolled in the course by academic advisors during summer orientation. Any first-year student who did not have an introductory course in the major (e.g., “Introduction to Education” or “Introduction to the Social Work Profession”) had the choice of signing up for one of 60 first-year seminar courses focused on a wide-range of topics. Five of these courses participated in health assessments and were included in the Seminar group (i.e., one section each of “Fight, Flight, or Freeze: The Impact of Stress”, “Freshman Wilderness Experience”, “MythBusters: Falcon Edition”, and two sections of “Live Well, Learn Well”). Among Seminar participants, informed consent was obtained in class near the end of the semester (week 12), which allowed for data collected as part of the course assignments (both in weeks 2–3 and in weeks 14–15) to be used for research purposes. These results were then compared to data collected from Health Study participants. Seminar students were not compensated for their participation. Study participants were not able to participate in both groups. The Bowling Green State University Institutional Review Board (Health Study: Project 342745, Approved June 23, 2012; Seminar: Project 936799, Approved August 18, 2016) approved both studies.

### 2.2. Measures

All measures were taken at baseline (second or third week of the semester) and at follow-up (14th or 15th week of the semester). Health measures included BP measured by an automated blood pressure cuff (Omron 5 Series, Omron Corporation, Kyoto, Japan), BMI calculated from measured height and weight, and %BF measured using bioelectrical impedance analysis (InBody 230; BioSpace, Seoul, Korea). Starting the Conversation (STC, a validated food frequency questionnaire) was used to assess changes in dietary behaviors [27]. The STC questionnaire includes eight questions regarding the intake of various types of foods over the past few months. The sum of the answers to these questions (each scored 0 = most, 2 = least healthful) provides a total diet quality score. A lower score indicates a more healthful diet (minimum = 0, maximum = 16) [27]. The STC questionnaire has successfully been used to identify changes in dietary patterns over a four-month period [27] (including with college students [28,29]), making it a useful measure of dietary behaviors in this timeframe and population. The Alcohol Use Disorders Identification Test-Consumption (AUDIT-C) was used to screen participants for heavy drinking or alcohol abuse [30]. The AUDIT-C has demonstrated excellent sensitivity in identifying heavy drinking as well as active alcohol dependence, and has been validated in college students [31]. The AUDIT-C consists of three questions, each scored 0 = least, 4 = most risky. The sum of the answers (minimum = 0, maximum = 12) provides a total risk score, with scores of 4 or more for males, and 3 or more for females, considered potentially hazardous [30].

### 2.3. Statistical Analysis

Paired sample *t*-tests, independent sample *t*-tests, Pearson’s Chi-square, Mann-Whitney U, and Wilcoxon Signed-Rank tests were used to compare changes over time and differences between groups. Analyses were completed using IBM SPSS Statistics, Version 24 (Armonk, New York, NY, USA). Results are presented as means ± standard error. The criterion for statistical significance in the data was *p* < 0.05, two tailed.

## 3. Results

A total of 191 participants (age: 18.1 ± 0.4 years) completed testing in the Health Study group, while 73 (age: 18.4 ± 0.5 years) of the 91 students (80%) in the Seminar group completed testing and also provided consent. In the Health Study group, 148 (77%) participants were female, while among the Seminar group, 40 (55%) were female. The sex distributions differed between studies (χ^2^ = 11.806, *p* = 0.001). Because of this difference and because sex differences have been reported for a variety of the measures of interest (e.g., %BF [32], body weight [33], or BP [34]), results from each study are presented by sex. A total of 30.2% of male Health Study participants indicated majoring in a health-related field (e.g., psychology, pre-nursing, dance) compared to 18.2% of male Seminar participants. Among females, 48.0% of Health Study and 48.8% of Seminar participants indicated majoring in a health-related field. The percentage of health-related majors did not differ between the groups (*p* > 0.05 for both). Sample sizes are provided in the tables and text and differ slightly among Seminar participants because several participants chose not to answer diet and alcohol questions.

### 3.1. Differences in Health Measures

In order to address the research question “do students who self-selected to participate in a health-based research study have more desirable health measures and health behaviors than students who completed health assessments as part of a first-year seminar course?”, baseline data between each study group were compared. In terms of health measures at baseline (Table 1), compared to males in the Health Study, diastolic BP was higher in male Seminar students (*p* = 0.006), while both systolic BP (*p* = 0.017) and diastolic BP (*p* < 0.001) were higher in female Seminar students. The percentage of Seminar participants who had undesirable BP (systolic ≥120 mmHg and/or diastolic ≥80 mmHg) was higher than Health Study participants for both males (*p* = 0.026) and females (*p* < 0.001). Female Seminar students weighed more than Health Study participants (*p* = 0.039). No differences in height were observed for either males or females. The percent of participants with an undesirable BMI was higher among Seminar females (*p* = 0.011). While BMI did not differ for the groups, the average BMI of Seminar males and females was considered overweight, while Health Study participants were classified as lean.

At follow-up (Table 1), both systolic and diastolic BP were higher among Seminar participants of both sexes (*p* ≤ 0.027 for all). The percentage of participants with undesirable BP was higher in female Seminar participants (*p* = 0.003). The percentage of participants with undesirable BMI was higher among Seminar females (*p* = 0.048).

In terms of absolute changes over time, males from both groups experienced an increase in weight and BMI, while males in the Seminar group also experienced an increase in %BF (Table 1; *p* ≤ 0.009 for all). Females in both groups experienced increases in weight and %BF (*p* ≤ 0.029 for all). BMI increased in Health Study females (*p* < 0.001). Females in the Health Study group experienced increases in systolic BP and in the percentage of participants with undesirable BP (*p* ≤ 0.003 for both). However, the percent change difference in these values did not differ between the groups.

### 3.2. Differences in Dietary Behaviors

In terms of dietary behavior at baseline, male Seminar participants (*N* = 29) indicated less-frequent added fat consumption compared to Health Study males (*N* = 43; *p* = 0.035; Table 2). Seminar females (*N* = 37) reported less frequent fruit and higher added fat consumption than Health Study females (*N* = 148, *p* ≤ 0.004 for both). No differences in total diet scores were observed at baseline, although Seminar participants had less-desirable scores.

At follow-up, Seminar males reported more frequent consumption of sweetened beverages compared to Health Study males (*p* = 0.022). While Seminar females reported less-frequent consumption of sweet snacks, consumption of lean protein was lower and added fat use was higher than Health Study females at follow-up (*p* ≤ 0.031 for all). Total diet score at follow-up was lower (more desirable) among Health Study females (*p* = 0.040); compared to baseline, female Seminar participants’ total diet scores worsened while Health Study participants’ scores improved, but the change in total diet score did not differ between groups.

### 3.3. Differences in Alcohol Consumption Patterns

At baseline, the pattern of responses differed between males in terms of amount consumed per day and binge drinking behaviors, with Seminar participants (*N* = 32) consuming more than Health Study participants (*N* = 43; Table 3). Female Seminar participants (*N* = 37) reported drinking more per day and more frequent binge drinking behaviors than Health Study participants (*N* = 148). At follow-up, male Seminar participants consumed alcohol more often, consumed more per day, and more frequently engaged in binge drinking behaviors than Health Study participants. Female Seminar students consumed more alcohol per day and engaged in more frequent binge drinking behaviors than Health Study participants. Drinking behaviors in males did not change over time. Health Study females increased the frequency of their drinking (*p* = 0.003) and binge drinking behaviors over time (*p* = 0.046). In terms of overall AUDIT-C scores, no differences were observed between the groups at either baseline or follow-up, although Seminar students had higher scores for all groups and at all time points. Only Health Study females’ total score increased over time (*p* = 0.008), but the follow-up score was lower than the baseline Seminar female score. Change in total alcohol score did not differ between any of the groups.

## 4. Discussion

This study compared the health measures and behaviors of participants who self-selected into a health-focused research study to students in an introductory seminar-style class. Identifying the magnitude of the effect of selection bias is difficult because the measurement of non-participants typically does not occur [35]. When differences in health measures and health behaviors were observed at baseline, these differences were almost always more undesirable among the Seminar participants, suggesting that participants who self-select into health-related studies are likely healthier than the general population. These findings agree with previous work that observed that participants who participated in a healthy aging study [14], a health check study [12], and an exercise study [11] were in better physical shape than those individuals who did not participate.

At follow-up, participants from the Health Study group had more desirable health measures than Seminar group participants. Health Study participants showed more evidence of improved dietary behaviors. Female Health Study participants did increase their use of alcohol over time; still, this increase was lower than Seminar participants’ use at baseline. While participants in the Seminar group might not have been as focused on their health as the participants who volunteered for the Health Study, participants in the Seminar group still had to agree to share their data, and we were unable to assess if the 20% of Seminar students who did not participate were significantly less healthy than those who did. Others have reported that individuals with increased adiposity as measured by waist-to-hip ratio were less likely to participate in follow-up study visits [16], and decreased response rates to an invitation to participate in a follow-up health examination were associated with higher initial BMI [18]. It is possible that if these data were available, it would result in further separation of the results between the groups, reflecting an even greater bias. Because students who enter their first year of university already classified as overweight or obese are at higher risk for developing chronic diseases [10], a better understanding of the magnitude of the health issues facing university students is of paramount importance.

In order to achieve samples that are less biased and more representative of the general university population, adequate compensation for participants may motivate more students who are not as health-conscious or healthy to participate. One report reviewed a number of studies that assessed participant motivation to enroll in health studies and noted that financial compensation was either the most important or a considerably important factor in determining participation [26]. How much participants should be paid is unknown. One study examined health study participant payment between 1997 and 1998, and found that participants were paid, on average, $9.50 per hour [21]. Adjusting for inflation using the U.S. Bureau of Labor Statistics Consumer Price Index Inflation Calculator [36], an equivalent payment in January 2016 would be $13.93 per hour. Given that our Health Study visits lasted less than 30 min, our payment of $5 or a spirit item (water bottle, T-shirt) did not exceed these suggestions. This likely means that our Health Study participants were very interested in monitoring their health over the course of the semester, as the compensation for participating was not especially generous. A variety of other recent (since 2012) studies have provided compensation in a variety of ways. Examples include: through course credit [37]; by allowing participants to choose 3 h of research credit or $20 per visit (equivalent of $6.66 per research credit hour) [38]; or did not report how participants were compensated [39]. Adequate compensation for these studies is an area that requires further examination.

There were distinct differences between groups at baseline and follow-up, differences in the degree of change between the time points were not routinely observed for absolute changes, and no percent change differences were noted for health measures between the groups. This was surprising, as there are a number of examples where self-selection has influenced study outcomes. For example, self-selection explained a significant portion of the relationship between lower BMI and the duration and rate of walking over a 7–10 year period [40]. That is, individuals who were lean at the beginning of the study chose to be more active than their heavier counterparts. Similarly, pre-exercise BMI was responsible for the relationship between fitness level, measured by running speed for a 10 km race and weekly running distance, and current BMI [41]. Discrepancies in the results of the present study compared to the results of these other studies might be attributed to the differences in the composition of the study populations (e.g., age, newly adjusting to the university environment, etc.). Still, the results from the present study suggest that, regardless of the motivation to self-select into a health-related study—which might or might not include an interest in one’s health—participants still experienced negative outcomes regarding anthropometric measures, and these changes occurred to the same degree as non-Health Study participants. It appears that motivation for study participation does not substantively influence changes in health outcomes in first-year university students.

### Strengths and Limitations

To our knowledge, this is the first attempt to assess whether self-selected volunteers who participate in first-year university student health studies differ significantly from the larger student population. While the repeated measures design increases the power to detect differences, sample sizes were still small, and the 4-month duration might not be long enough to see all changes. Self-reported diet and alcohol measures are subject to under-reporting, and the small pool of male participants means that generalization of results should be made with caution when comparing between groups. While Seminar participants did not deliberately decide to participate in a health study, they were able to decide whether or not they would share their data, so the differences we observed likely underestimate true differences based on the previously cited work suggesting differences between participants and non-participants.

## 5. Conclusions

Based on these results, students who self-select into health-related studies typically start off and end up with better health indicators but experience changes similar to those students who do not enroll in these types of studies. We conclude that health studies among university students that use participants who self-select likely underestimate the prevalence of undesirable health measures and behaviors, but that these studies likely accurately reflect changes that occur over time in the larger first-year university student population.

## Figures and Tables

**Table 1:** Health measures.

	Health Study (*n* = 43 Male, 148 Female)				Seminar (*n* = 30 Male, 40 Female)			
	Baseline	Follow-Up	Absolute Change	Percent Change	Baseline	Follow-Up	Absolute Change	Percent Change
**Systolic BP (mmHg)**								
Male ^d^	124.0 ± 2.4	128.3 ± 1.6	4.3 ± 2.2	5.6 ± 3.2%	129.3 ± 2.1	134.7 ± 2.5	5.4 ± 3.3	5.0 ± 2.7%
Female ^a,c,d^	114.9 ± 0.8	118.3 ± 0.9	3.4 ± 0.9	3.4 ± 0.8%	121.2 ± 2.4	122.8 ± 2.2	1.6 ± 2.0	2.1 ± 1.7%
**Diastolic BP (mmHg)**								
Male ^c,d^	71.9 ± 1.7	71.7 ± 1.8	−0.2 ± 2.0	0.9 ± 2.6%	79.4 ± 1.8	81.4 ± 1.7	2.4 ± 2.2	4.5 ± 2.7%
Female ^c,d^	69.5 ± 0.6	70.4 ± 0.9	0.9 ± 1.0	2.0 ± 1.4%	79.4 ± 1.8	81.7 ± 1.3	2.2 ± 1.8	4.4 ± 2.6%
**Undesirable BP**								
Male ^c^	69.8%	74.4%	4.6%	6.6%	93.3%	93.3%	0.0%	0.0%
Female ^a,c,d^	34.5%	46.6%	12.2%	34.8%	64.1%	74.4%	10.3%	16.1%
**Height (cm)**								
Male	177.6 ± 0.9	177.9 ± 1.0	0.3 ± 0.1	0.2 ± 0.1%	178.7 ± 1.4	179.1 ± 1.4	0.4 ± 0.3	0.2 ± 0.2%
Female	163.2 ± 0.5	163.4 ± 0.5	0.2 ± 0.1	0.1 ± 0.0%	164.8 ± 0.8	165.3 ± 0.8	0.4 ± 0.1	0.2 ± 0.1%
**Weight (kg)**								
Male ^a,b^	74.2 ± 2.1	75.8 ± 2.1	1.6 ± 0.3	2.2 ± 0.4%	80.4 ± 3.8	82.5 ± 3.8	2.0 ± 0.6	2.7 ± 0.7%
Female ^a,b,c^	62.7 ± 1.3	64.1 ± 1.3	1.4 ± 0.2	2.2 ± 0.3%	70.5 ± 3.4	71.7 ± 3.5	0.9 ± 0.3	1.5 ± 0.6%
**BMI (kg/m^2^)**								
Male ^a,b^	23.5 ± 0.6	23.9 ± 0.6	0.4 ± 0.1	1.5 ± 0.4%	25.1 ± 1.1	25.6 ± 1.1	0.5 ± 0.2	2.2 ± 0.8%
Female ^a^	23.5 ± 0.4	23.9 ± 0.4	0.4 ± 0.1	1.9 ± 0.3%	26.1 ± 1.3	26.2 ± 1.2	0.2 ± 0.2	0.9 ± 0.7%
**Undesirable BMI**								
Male	27.9%	30.2%	2.3%	8.2%	37.5%	40.6%	3.1%	8.3%
Female ^c,d^	21.6%	26.4%	4.8%	22.2%	41.5%	41.5%	0.0%	0.0%
**Body Fat (%)**								
Male ^b^	15.2 ± 1.2	16.6 ± 1.0	1.4 ± 0.4	15.7 ± 3.4%	16.5 ± 1.6	18.0 ± 1.7	1.6 ± 0.5	13.3 ± 4.8%
Female ^a,b^	28.5 ± 0.7	29.5 ± 0.6	1.1 ± 0.3	8.7 ± 4.5%	30.1 ± 1.7	31.7 ± 1.7	1.6 ± 0.4	7.9 ± 2.6%

All values are means ± standard error of the mean. Abbreviations: BP = blood pressure, BMI = body mass index. Absolute change = follow-up − baseline. Percent change = absolute change/baseline. Classifications: Undesirable BP = systolic ≥120 mmHg and/or diastolic ≥80 mmHg. Undesirable BMI ≥25 kg/m^2^. Significant differences (*p* < 0.05) are indicated by superscripts: ^a^ for absolute changes over time in the Health Study group, ^b^ for absolute changes over time in the Seminar group, ^c^ for differences at baseline between groups, and ^d^ for difference at follow-up between groups.

**Table 2:** Diet behavior.

	Health Study						Seminar					
	Most Healthful		Somewhat Healthful		Least Healthful		Most Healthful		Somewhat Healthful		Least Healthful	
	Base-line	Follow-up	Base-line	Follow-up	Base-line	Follow-up	Base-line	Follow-up	Base-line	Follow-up	Base-line	Follow-up
**How many times a week did you eat fast food meals or snacks?**												
	<1		1 to 3		4+		<1		1 to 3		4+	
Male	30.2	34.9	48.8	55.8	20.9	9.3	24.1	43.5	58.6	43.5	17.2	13.0
Female ^a^	21.6	34.5	52.7	54.7	25.7	10.8	27.0	34.4	59.5	50.0	13.5	15.6
**How many servings of fruit did you eat each day?**												
	5+		3 to 4		≤2		5+		3 to 4		≤2	
Male	4.7	0.0	53.5	51.2	41.9	48.8	6.9	4.3	34.5	52.2	58.6	43.5
Female ^a,b^	10.2	3.4	41.5	41.9	48.3	54.7	0.0	0.0	24.3	28.1	75.7	71.9
**How many servings of vegetables did you eat each day?**												
	5+		3 to 4		≤2		5+		3 to 4		≤2	
Male	9.3	7.0	41.9	39.5	48.8	53.5	10.3	8.7	17.0	43.5	72.4	47.8
Female	2.7	5.4	41.2	33.1	56.1	61.5	0.0	0.0	35.1	28.1	64.9	71.9
**How many regular sodas or glasses of sweet tea did you drink each day?**												
	<1		1 to 2		3+		<1		1 to 2		3+	
Male ^c^	55.8	79.1	30.2	18.6	14.0	2.3	37.9	52.2	55.2	39.1	6.9	8.7
Female	61.5	73.6	23.6	20.3	14.9	6.1	64.9	59.4	29.7	34.4	5.4	6.3
**How many times a week did you eat beans (like pinto or black beans), chicken, or fish?**												
	3+		1 to 2		<1		3+		1 to 2		<1	
Male	72.1	58.1	20.9	34.9	7.0	7.0	55.2	56.5	34.5	26.1	10.3	17.4
Female ^c^	60.1	52.0	29.7	37.2	10.1	10.8	45.9	28.1	32.4	43.8	21.6	28.1
**How many times a week did you eat regular snack chips or crackers (not low-fat)?**												
	≤1		2 to 3		4+		≤1		2 to 3		4+	
Male	55.8	69.8	34.9	20.9	9.3	9.3	44.8	52.2	44.8	43.5	10.3	4.3
Female ^c^	52.0	52.7	39.9	41.2	8.1	6.1	40.5	34.4	45.9	40.6	13.5	25.0
**How many times a week did you eat desserts and other sweets (not the low-fat kind)?**												
	≤1		2 to 3		4 +		≤1		2 to 3		4+	
Male	23.3	20.9	51.2	58.1	25.6	20.9	24.1	39.1	72.4	43.5	3.4	17.4
Female ^a,c^	27.7	14.2	51.4	52.0	20.9	33.8	18.9	28.1	64.9	53.1	16.2	18.8
**How much margarine, butter, or meat fat do you use to season vegetables or put on potatoes, bread, or corn?**												
	≤1		2 to 3		4+		≤1		2 to 3		4+	
Male ^a,b^	51.2	76.7	39.5	18.6	9.3	4.7	75.9	73.9	20.7	26.1	3.4	0.0
Female ^a,b^	41.2	61.5	50.0	35.8	8.8	2.7	67.6	59.4	29.7	40.6	2.7	0.0
**Starting the Conversation Food Frequency Questionnaire Summary Score**												
	Baseline			Follow-Up			Baseline			Follow-Up		
Male	6.8 ± 0.4			6.1 ± 0.4			7.0 ± 0.5			6.2 ± 0.7		
Female ^c^	7.2 ± 0.2			6.9 ± 0.2			7.5 ± 0.4			7.9 ± 0.4		

All values are percentages. Significant differences (*p* < 0.05) are indicated by superscripts: ^a^ for changes over time in the Health Study group, ^b^ for differences at baseline between groups, and ^c^ for difference at follow-up between groups.

**Table 3:** Alcohol.

**How Often Do You Have a Drink Containing Alcohol? (Frequency)**							
**Male**		Never	Monthly or less	2–4 times per month	2–3 times per week	4+ times per week	*p*-value
Health Study	Baseline	58.1	27.9	11.6	2.3	0.0	N.S.
Seminar	Baseline	44.8	20.7	27.6	6.9	0.0	
Health Study	Follow-Up	62.8	20.9	16.3	0.0	0.0	*p* = 0.049
Seminar	Follow-Up	45.5	9.1	36.4	9.1	0.0	
**Female ^a^**							
Health Study	Baseline	56.1	27.0	16.2	0.7	0.0	N.S.
Seminar	Baseline	48.6	29.7	13.5	8.1	0.0	
Health Study	Follow-Up	49.3	26.4	18.9	4.1	1.4	N.S.
Seminar	Follow-Up	39.4	30.3	27.3	0.0	3.0	
**How many standard drinks containing alcohol do you have on a typical day? (Amount)**							
**Male**		1 or 2	3 or 4	5 or 6	7 to 9	10+	
Health Study	Baseline	76.7	16.3	7.0	0.0	0.0	*p* < 0.001
Seminar	Baseline	25.0	18.8	37.5	18.8	0.0	
Health Study	Follow-Up	74.4	14.0	11.6	0.0	0.0	*p* = 0.023
Seminar	Follow-Up	18.2	18.2	36.4	18.2	9.1	
**Female**							
Health Study	Baseline	83.8	10.8	2.7	1.4	1.4	*p* < 0.001
Seminar	Baseline	63.2	15.8	15.8	5.3	0.0	
Health Study	Follow-Up	79.7	11.5	5.4	3.4	0.0	*p* = 0.022
Seminar	Follow-Up	55.0	30.0	15.0	0.0	0.0	
**How often do you have six or more drinks on one occasion? (Binge)**							
**Male**		Never	Less than monthly	Monthly	Weekly	Daily or almost daily	
Health Study	Baseline	74.4	18.6	4.7	2.3	0.0	*p* < 0.001
Seminar	Baseline	25.0	31.3	25.0	18.8	0.0	
Health Study	Follow-Up	81.4	9.3	7.0	2.3	0.0	*p* < 0.001
Seminar	Follow-Up	9.1	36.4	45.5	9.1	0.0	
**Female ^a^**							
Health Study	Baseline	87.2	8.8	2.0	2.0	0.0	*p* < 0.001
Seminar	Baseline	42.1	47.4	0.0	10.5	0.0	
Health Study	Follow-Up	82.4	9.5	6.1	2.0	0.0	*p* < 0.001
Seminar	Follow-Up	35.0	50.0	10.0	5.0	0.0	
**Total AUDIT-C Score**							
**Male**				**Female ^a^**			
Health Study	Baseline	1.2 ± 0.3	N.S.	Health Study	Baseline	1.1 ± 0.2	N.S.
Seminar	Baseline	2.6 ± 0.6		Seminar	Baseline	1.5 ± 0.3	
Health Study	Follow-Up	1.2 ± 0.3	N.S.	Health Study	Follow-Up	1.4 ± 0.2	N.S.
Seminar	Follow-Up	2.8 ± 0.7		Seminar	Follow-Up	1.9 ± 0.4	

All values are percentages. *p*-values in the table indicate differences between groups at baseline and at follow-up. Significant differences (*p* < 0.05) over time are indicated by superscripts: ^a^ for changes over time in the Health Study group.
